# Local infiltration anesthesia (anterior field block) vs. general anesthesia for thyroidectomy: effects on operative time, complications, and cost—a GRADE-assessed systematic review and meta-analysis with subgroup analysis

**DOI:** 10.3389/fsurg.2026.1697921

**Published:** 2026-09-30

**Authors:** Khadeeja Ali Hamzah, Hassan H. Eladl, Shehab Elfiqi, Ahmed Abdelkader Saad Elsehsah, Amr E. Zeweta, Ahmed Nabil Ali, Mohamed Wagdy, Yousif Hameed Kurmasha, Yasmeen Jamal Alabdallat

**Affiliations:** 1 Al-Kindy College of Medicine, University of Baghdad, Baghdad, Iraq; 2 Medical Research Group of Egypt (MRGE), Negida Academy LLC, Arlington, MA, United States; 3 Faculty of Medicine, Ain Shams University, Cairo, Egypt; 4 Faculty of Medicine, Modern University for Technology and Information, Cairo, Egypt; 5 College of Medicine, University of Kufa, Najaf, Iraq; 6 Hashemite University, Zarqaa, Jordan

**Keywords:** general anesthesia, hospital stay, local anesthesia, operative time, thyroidectomy

## Abstract

**Introduction:**

Thyroidectomy involves the removal of part or all of the thyroid gland, commonly performed for goiters, hyperthyroidism, or carcinomas, with 450,000–500,000 procedures performed annually in the United States. General anesthesia is traditionally used, providing optimal surgical conditions but associated with risks such as cardiovascular complications and longer hospital stays. Local anesthesia is gaining interest for high-risk patients, offering potentially similar outcomes to general anesthesia, while reducing postoperative complications and costs. The choice of anesthetic technique impacts patient safety, healthcare costs, and overall experience, yet no meta-analysis has previously compared local and general anesthesia for thyroidectomy.

**Aim:**

This systematic review and meta-analysis aimed to compare surgical and perioperative outcomes of general anesthesia vs. local anesthesia in thyroidectomy, focusing on operative time, complications, cost, and postoperative recovery, to inform clinical practice.

**Methods:**

This review was conducted according to Preferred Reporting Items for Systematic Reviews and Meta-Analyses guidelines. A systematic search of PubMed/MEDLINE, Embase, Web of Science, and Scopus was performed from database inception to August 2025. We included randomized controlled trials, cohort studies, and case–control studies comparing local infiltration anesthesia with general anesthesia in patients with thyroidectomy. Key outcomes included operative time, intraoperative and postoperative complications, hospital stay length, pain scores, patient satisfaction, and cost. Data were extracted independently by two authors. Risk of bias was assessed using the Cochrane Risk of Bias 2 (RoB 2) tool for randomized trials and the Risk of Bias In Non-Randomized Studies of Interventions tool for non-randomized studies. Certainty of evidence was evaluated using the Grading of Recommendations Assessment, Development and Evaluation framework. Statistical analysis was performed using Review Manager (RevMan), calculating mean differences and risk ratios under a random-effects model, with heterogeneity assessed using the *I*^2^ statistic. Statistical significance was set at *p* < 0.05.

**Results:**

The systematic search identified 1,785 studies, of which 252 duplicates were removed. After screening, 1,508 studies were excluded, leading to a full-text review of 25 studies and ultimate inclusion of 5 studies comprising 508 patients undergoing thyroid surgery with either local or general anesthesia. Local anesthesia was associated with a shorter operative time than general anesthesia (mean difference −18.32 min, 95% CI: −27.31 to −9.15), but heterogeneity was high (*I*^2^ = 95%), and the effect was significant only in cohort studies and not in randomized trials, and therefore, this finding should be interpreted with caution. No significant differences were observed between groups in nerve injury or respiratory distress, although evidence certainty for these outcomes was low. A cost analysis suggested that local anesthesia was less expensive than general anesthesia across all studies reporting cost data. Patient satisfaction appeared comparably high in both groups across the studies that assessed this factor.

**Conclusion:**

Local anesthesia was associated with shorter operative times, lower costs, and shorter hospital stays compared with general anesthesia in thyroidectomy. However, these findings are drawn from a small number of studies and therefore should be interpreted cautiously. General anesthesia also likely remains necessary for more advanced procedures requiring airway control and complete patient immobility.

## 1 Introduction

Thyroidectomy is a surgical procedure involving the removal of part or all of the thyroid gland, most commonly performed for goiters, hyperthyroidism, or thyroid carcinoma (1–3). Thyroid disorders are among the most prevalent endocrine conditions worldwide, affecting an estimated 200 million people globally (4, 5), and thyroid cancer is the most rapidly increasing cancer diagnosis internationally, with approximately 586,000 new cases reported worldwide in 2020, making it the ninth most common cancer globally (6). Procedure volumes vary considerably by country and healthcare system: an estimated 450,000–500,000 thyroidectomies are performed annually in the United States alone (7, 8), while European data report more than 30,000 annually in Poland, 45,000 in France, 60,000 in Germany, and over 4,000 in Austria (9). Regardless of region, the high and rising global volume of thyroid surgery means that optimizing perioperative care has a substantial impact on patient outcomes and healthcare systems.

The choice of anesthetic strategy is one of the most important factors in thyroid surgery (10, 11). General anesthesia (GA) has traditionally been the standard approach, offering complete unconsciousness, muscle relaxation, and airway control, which together provide optimal surgical conditions and allow prolonged procedures to be performed without patient discomfort (12, 13). However, general anesthesia is also associated with perioperative risks such as cardiovascular and respiratory complications, particularly in elderly patients, as well as longer hospital stays and higher costs (14).

Over the past decade, there has been increasing interest in local anesthesia (LA), particularly for patients at higher surgical risk (15). This interest is driven by the potential of local anesthesia to reduce postoperative nausea and vomiting, enable faster recovery, and avoid complications associated with general anesthesia and endotracheal intubation (16). It has also been suggested that local anesthesia allows for early detection of recurrent laryngeal nerve (RLN) injury through intraoperative patient vocalization, an assessment not possible under general anesthesia (11, 17). Recent studies indicate that local anesthesia may achieve surgical outcomes similar to general anesthesia, while reducing the need for intensive postoperative care and lowering overall costs (18).

The choice between these two anesthetic approaches has meaningful implications across several domains. Patient safety considerations include airway management and hemodynamic stability, both of which differ substantially between techniques. Postoperative pain, nausea, cognitive function, and general wellbeing have also been reported to differ between anesthetic techniques, directly shaping patient experience (8, 19). These clinical differences carry economic consequences as well: variation in operating room efficiency, recovery time, length of hospital stay, and resource use directly affects healthcare costs.

Despite the clinical relevance of this decision and a growing body of individual studies, no meta-analysis to date has quantitatively compared local and general anesthesia in thyroidectomy, leaving clinicians without pooled, quality-assessed evidence to guide anesthetic selection—particularly for patients who may be considered higher-risk candidates for general anesthesia. This systematic review and meta-analysis addresses this gap by pooling available comparative evidence on operative time, complications, cost, patient satisfaction, and postoperative recovery, with the aim of providing clinicians with a clearer, evidence-based basis for anesthetic choice in thyroid surgery.

## 2 Methods

This review was not prospectively registered, and this is acknowledged as a limitation. This review followed the Preferred Reporting Items for Systematic Reviews and Meta-Analyses (PRISMA) statement guidelines (20).

### 2.1 Eligibility criteria

We included randomized controlled trials (RCTs), cohort studies, and case–control studies involving patients undergoing thyroidectomy for benign or malignant thyroid conditions. Eligible studies compared local infiltration anesthesia with general anesthesia and reported at least one relevant outcome measure. In this review, local infiltration anesthesia refers specifically to local lidocaine infiltration as an anterior field block combined with monitored anesthesia care (MAC), which additionally involves intravenous sedation.

We excluded case reports, reviews, expert opinions, and studies lacking a direct comparison between local and general anesthesia. Studies not published in English were also excluded.

### 2.2 Outcomes

Our primary outcome was operative time, defined as the duration from a patient's entry into the operating room until entry into the recovery room, measured in minutes. Operative time was selected as the primary outcome because it directly reflects operating room efficiency and resource utilization, which have substantial downstream effects on healthcare costs, staffing burden, and system-level capacity, particularly relevant given the high global volume of thyroidectomy procedures.

Secondary outcomes were as follows: length of hospital stay; postoperative pain scores and analgesic use; patient satisfaction and quality of life; intraoperative complications, including bleeding, hematoma, and pain score; nerve injury; respiratory distress; hypoparathyroidism; postoperative complications, including hematoma, hypocalcemia, and infection; and cost.

### 2.3 Search strategy

We conducted a systematic literature search across four electronic databases—PubMed/MEDLINE, Embase, Web of Science, and Scopus—from inception to August 2025. The search was limited to English-language studies. No restriction was placed on publication date, geographic region, or healthcare setting. The following search terms were used: (“thyroidectomy” OR “thyroid surgery”) AND (“local anesthesia” OR “regional anesthesia” OR “cervical block”) AND (“general anesthesia”). The full search strategy is provided in the Supplementary material.

### 2.4 Selection process

Study selection was performed manually using Rayyan in two sequential steps: title/abstract screening followed by full-text screening. Screening at both stages was performed independently by two authors. Conflicts were resolved through a discussion between the two screening authors, with a third independent reviewer consulted in cases where consensus could not be reached.

### 2.5 Data extraction

Two independent authors extracted data on study characteristics, including the following: study design, first author and year, study period, country, sample size per treatment arm, patient age, sex distribution, surgical indication (e.g., benign disease and malignancy), extent of thyroidectomy performed, anesthetic protocol details for each arm, and a summary of reported results. Data related to the specific outcomes of interest, such as operative time, incidence of hypoparathyroidism, nerve injury, respiratory distress, and hospital stay, were extracted in parallel. Where outcomes were reported as median and interquartile range (IQR), these were converted to mean and standard deviation (SD) using the method described by Wan et al. (21). Costs reported in Indian Rupees by individual studies were converted to US Dollars (USD) using the average annual exchange rate for the year of each study's publication (44.00 INR/USD for 2005; 65.07 INR/USD for 2017), as reported in the US Federal Reserve's G.5A release, to enable descriptive comparison across studies; these are study-level costs converted individually and not a pooled mean calculated across currencies, and original currency values are retained alongside USD equivalents in Table 1.

**Table 1:** Baseline characteristics of the study population.

Study ID	Anesthesia	*N* (M/total)	Age mean (range)	Benign: solitary	Benign: multinodular	Benign: colloid	Malignant: papillary	Malignant: follicular	Lesion size (cm)	Hemithyroidectomy	Subtotal	Total	Operative time (min)	Cost, mean	Notes
Shukla (33)	LA-MAC	7/50	33.3 (18–55)	38	12	8	4	NR	5.26 (2.3–7.9)	19	16	15	41.6	Rs. 2189.32 (US $49.76)	26% discharged <24 h
	GA	14/50	38.4 (25–68)	32	18	13	5	NR	6.33 (3.6–10.1)	17	11	22	74.5	Rs. 5520.00 (US $125.45)	
Snyder et al. (16)	LA-MAC	3/29	52.1 ± 16.7	NR	15	NR	3	NR	NR (wt: 37 g)	NR	NR	29^a^	159 ± 29	$315 savings vs GA	Claustrophobia exclusion
	GA	2/29	46.9 ± 15.8	NR	14	NR	5	NR	NR (wt: 26 g)	NR	NR	29^a^	163 ± 37	Reference	
Chintale et al. (32)	LA-MAC	6/100	34.5 (20–65)	50	15	25	5	5	4.5 (2.3–8.5)	75	15	20	50.5 ± 6.04	Rs. 2500 (US $38.42)	
	GA	8/100	37.5 (18–65)	30	50	10	6	4	6.5 (4.5–12)	60	30	10	75.5 ± 7.8	Rs. 5500 (US $84.52)	
Kim (38)	LA-MAC	9/25	48 ± 15.1	NR	NR	NR	24	NR	NR	NR	NR	25	112.17 ± 18.01	NR	Predominantly PTC
	GA	8/25	50 ± 15.1	NR	NR	NR	NR	NR	NR	NR	NR	25	106.89 ± 26.38	NR	
Kabuye et al. (8)	LA-MAC	2/29	39.3 ± 14.1	NR	NR	NR	NR	NR	NR	9 (31.0%)	NR	20 (69.0%)	NR^b^	64.5 USD	Grade 1–2 goiter only
	GA	3/29	43.5 ± 10.0	NR	NR	NR	NR	NR	NR	9 (31.0%)	NR	20 (69.0%)	NR^b^	76.6 USD	

LA-MAC, local anesthesia with monitored anesthesia care; GA, general anesthesia; *N*, number of participants; M, male; NR, not reported; PTC, papillary thyroid cancer.

None of the five included studies reported IONM use or formally assessed perioperative psychological anxiety.

a Operative time = total duration from patient entry into the operating room until entry into the recovery room (per Section 2.2), encompassing anesthetic preparation, surgical draping, the procedure, and wound closure.

b Kabuye et al. (8) did not report operative time data and was excluded from the pooled operative time meta-analysis.

### 2.6 Risk of bias

Two authors independently assessed the risk of bias (ROB) of the included RCTs using the Cochrane Risk of Bias 2 (RoB 2) tool (22), evaluating the following domains: bias arising from the randomization process, bias due to deviations from intended interventions, bias due to missing outcome data, bias in measurement of the outcome, bias in selection of the reported result, and overall risk of bias. Conflicts were resolved by discussion. Non-randomized studies were assessed using the Risk of Bias in Non-randomized Studies of Interventions (ROBINS-I) tool (23), evaluating the following: confounding, selection of participants into the study, classification of interventions, deviation from intended intervention, missing data, measurement of outcomes, selection of the reported result, and overall bias. Risk-of-bias judgments are represented as follows: for RoB 2 (RCTs), green/“+” indicates low risk, yellow/“!” indicates some concerns, and red/“−” indicates high risk of bias. For ROBINS-I (cohort studies), green indicates low risk, yellow indicates moderate risk, and red indicates serious or critical risk of bias.

### 2.7 Quality of the evidence

Certainty of evidence for each outcome was appraised using the Grading of Recommendations Assessment, Development and Evaluation (GRADE) framework (24, 25). Two reviewers independently assessed certainty across five domains: risk of bias, inconsistency, indirectness, imprecision, and publication bias. Formal assessment of publication bias using funnel plot asymmetry testing was not performed given the small number of included studies (*n* = 5), as a minimum of ten studies is conventionally required for reliable interpretation (26); Cochrane Handbook for Systematic Reviews of Interventions, Chapter 13 (27). Instead, the possibility of publication bias was considered qualitatively within the GRADE framework as part of the overall certainty assessment and is noted as a limitation where applicable. Evidence was rated as high, moderate, low, or very low according to GRADE classification. Conflicts between reviewers on GRADE domain ratings were resolved by discussion, with consensus serving as the final judgment.

### 2.8 Statistical analysis

Statistical analysis was performed using RevMan. For continuous outcomes, mean differences (MD) with 95% confidence intervals (CIs) were calculated using a random-effects model. For dichotomous outcomes, risk ratios (RR) with 95% CI were computed. Heterogeneity was assessed using the *I^2^* statistic, with values of 25%, 50%, and 75% representing low, moderate, and high heterogeneity, respectively. Statistical significance was set at *p* < 0.05. A subgroup analysis was performed based on study design (RCTs vs. cohort studies) to explore sources of heterogeneity. Forest plots were generated to visually represent effect sizes and confidence intervals for each outcome. A leave-one-out sensitivity analysis was performed for the primary outcome (operative time) by sequentially excluding each contributing study and recalculating the pooled random-effects estimate, to assess the influence of individual studies on the overall effect and heterogeneity. Publication bias assessment was not performed because of the limited number of included studies (*n* = 5).

## 3 Results

### 3.1 Study selection

A systematic search identified 1,785 studies, with 252 duplicates removed. After screening the titles and abstracts of 1,533 studies, 1,508 were excluded for irrelevance or non-comparative study design. A full-text review was conducted on 25 studies, leading to the exclusion of 20 studies due to irrelevant comparison or inappropriate study design. Ultimately, five studies were included in the quantitative and qualitative analysis. This is illustrated in the PRISMA flow chart (Figure 1).

**Figure 1:**
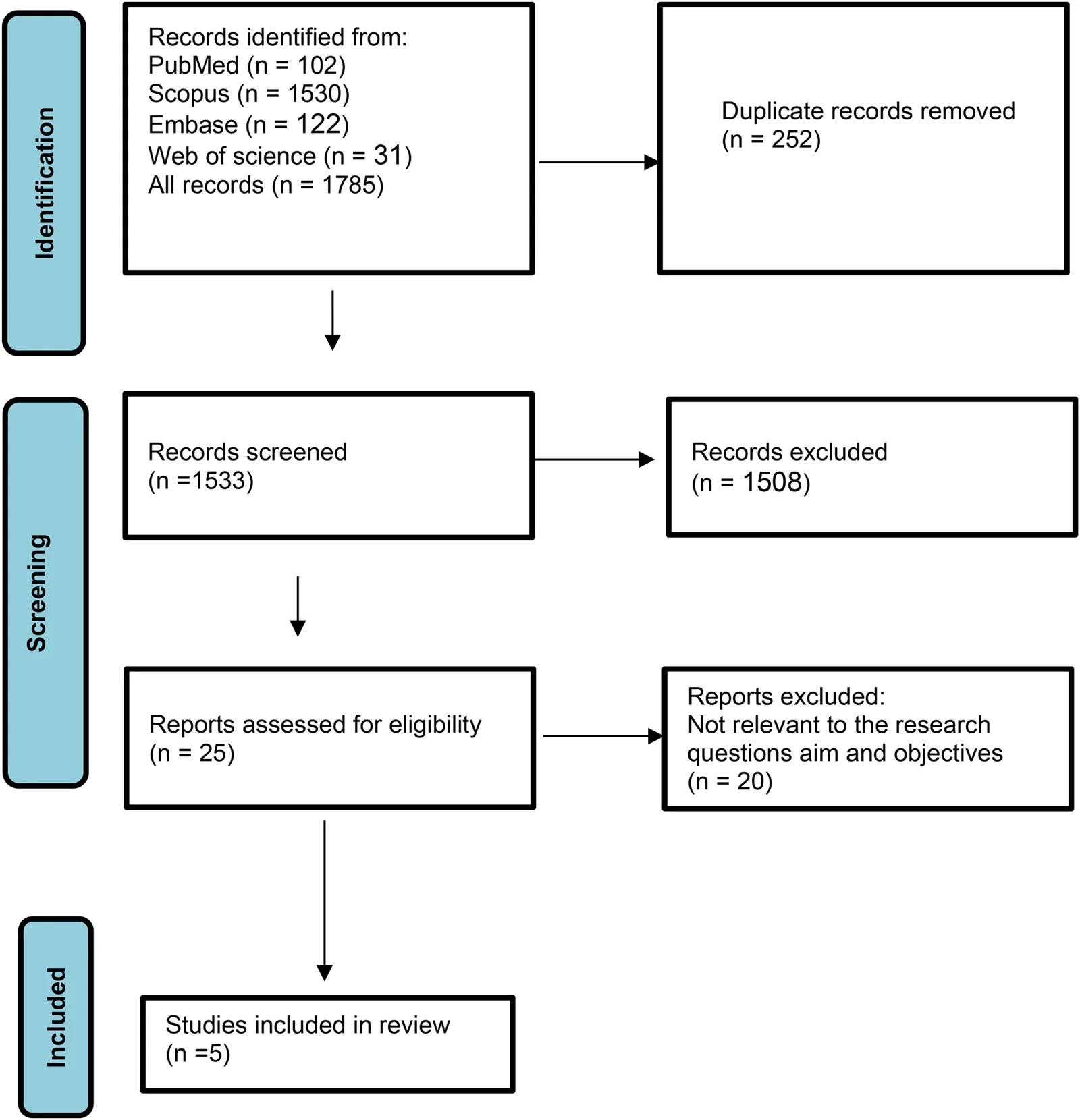
A PRISMA flow diagram.

### 3.2 Summary of included studies

A total of five studies, comprising three cohort studies and two RCTs, were included in this meta-analysis, involving 508 patients undergoing thyroid surgery under either local or general anesthesia. The studies were conducted across four regions: the United States, India, Republic of Korea, and Uganda, with publication dates ranging from 2005 to 2025 (Table 1). The included studies were Shukla (33), Snyder et al. (16), Chintale et al. (32), Kim (38), and Kabuye et al. (8). Baseline characteristics of the study populations are summarized in Table 2.

**Table 2:** A summary of included studies.

Study ID	Design	Region	Duration	*N* (LA)	*N* (GA)	Total	Inclusion criteria	Primary outcome(s)	Secondary outcome(s)	Key results
Shukla (33)	Cohort	India	Jan 1996–Sep 2003	50	50	100	All patients undergoing thyroid surgery for benign and malignant thyroid disease	**Operative time**	Respiratory distress Mean analgesia requirement Surgical complications Cost Hospital stay Nerve injury Hypothyroidism	The authors reported a shorter operative time, lower cost, and shorter hospital stay with LA compared with GA. Complication rates were low in both groups (6% LA vs. 4% GA), and 26% of LA patients were discharged within 24 h.
Snyder et al. (16)	RCT	United States	1996–2003	50	50	100	Patients undergoing thyroid surgery under local or general anesthesia	**Operative time**^a^ Patient satisfaction Cost	Hospital admission Postoperative complications Hypothyroidism Patient satisfaction	No significant differences were found in demographics, postoperative adverse symptoms, complications, or patient satisfaction. The GA group spent significantly more time postoperatively until same-day discharge (*p* = 0.02).
Chintale et al. (32)	Cohort	India	Mar 2014–Mar 2017	100	100	200	All patients undergoing thyroid surgery (benign or malignant thyroid disease)	**Operative time** Cost-effectiveness	Nerve injury Respiratory distress Hospital stay and complications	Local anesthesia was described as a safe alternative to general anesthesia for patients undergoing thyroid surgery.
Kim (38)	RCT	Republic of Korea	Dec 2015–Jul 2016	25	25	50	Age ≥18 years; thyroidectomy or selective neck dissection; preoperative PTC confirmed by US-guided FNA cytology	Postoperative discomfort (nausea, vomiting, pain, throat discomfort, voice changes)	Operative time Patient satisfaction Nerve injury	The authors reported comparable patient satisfaction between the two groups, with fewer postoperative nausea, vomiting, throat discomfort, and voice changes in the LA-MAC group compared with GA.
Kabuye et al. (8)	Cohort	Uganda	Oct 2022–Apr 2023	29	29	58	Euthyroid patients aged 18–65; grade 1 and 2 goiter; consented to study	Early postoperative outcomes: nausea, vomiting, hematoma, transient voice changes, pain	Nerve injury Respiratory distress Material and medication costs Patient satisfaction	No significant differences were found in demographics, symptom duration, early postoperative complications, or patient satisfaction (*P* > 0.05).

LA, local anesthesia; GA, general anesthesia; RCT, randomized controlled trial; PTC, papillary thyroid cancer; FNA, fine needle aspiration; *N*, number of participants.

Bolded outcomes indicate those pooled in this review's primary meta-analysis (operative time; Figures 2, 3).

a Snyder et al. (16) reports this outcome as “time in surgical care stages” in the original study; it corresponds to this review's operative time outcome and was pooled accordingly in Figure 2.

### 3.3 Primary outcome

Operative time, defined as the duration from a patient's entry into the operating room until entry into the recovery room, was reported in four studies [Shukla (33), Snyder et al. (16), Chintale et al. (32), Kim (38)]. The pooled analysis estimated a shorter operative time with local anesthesia compared with general anesthesia (MD −18.32 min, 95% CI: −27.31 to −9.15, *p* < 0.0001; Figure 2); however, heterogeneity across pooled studies was substantial (*I*^2^ = 95%, *p* < 0.0001), and this pooled estimate should be interpreted with considerable caution.

**Figure 2:**
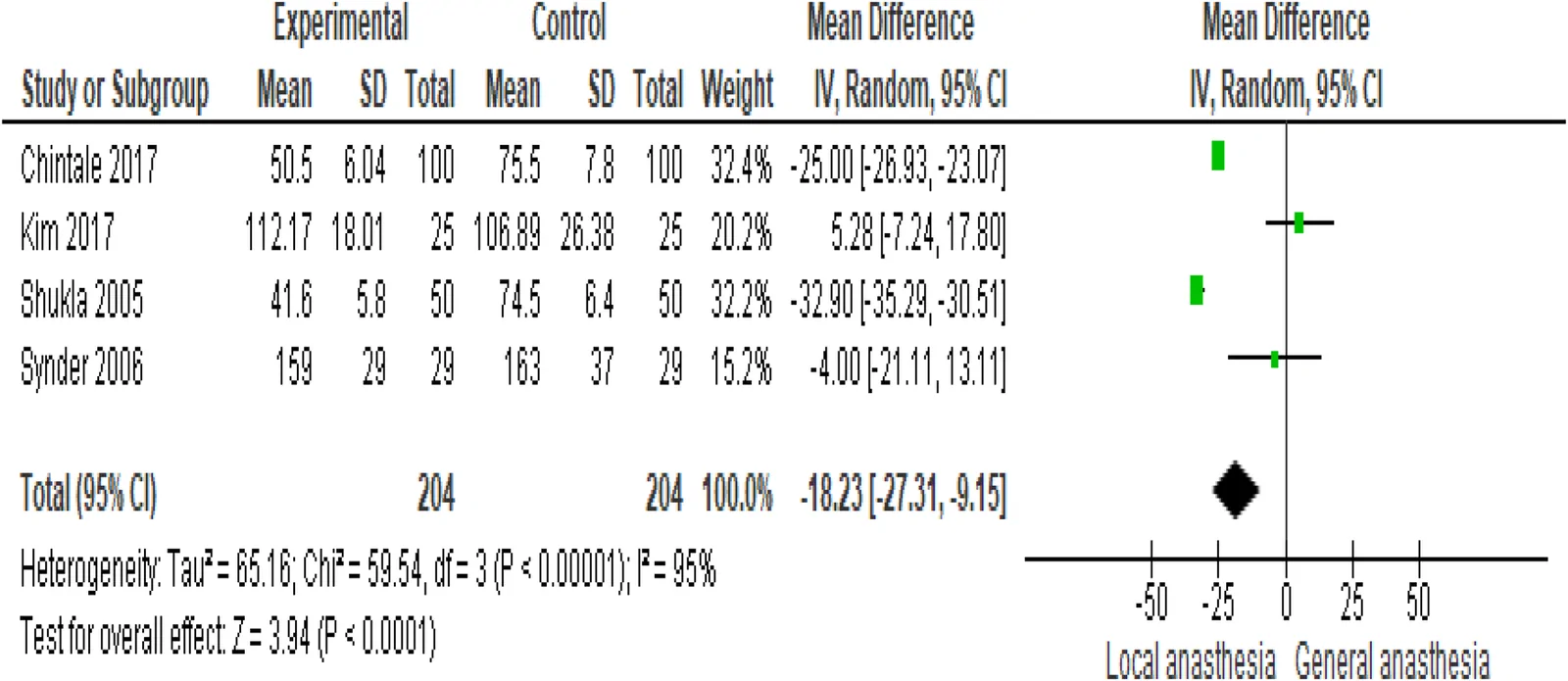
A forest plot of OR in minutes.

A subgroup analysis by study design revealed a marked discordance between RCTs and cohort studies (Figure 3). Among the two RCTs, the pooled effect was not statistically significant and favored general anesthesia (MD 2.04 min, 95% CI: −8.06 to 12.15, *p* = 0.69; *I*^2^ = 0%), with the two contributing trials showing opposing point estimates [Kim (38): MD +5.28; Snyder et al. (16): MD −4.00] on either side of the null. In contrast, the cohort studies showed a large, statistically significant effect favoring local anesthesia (MD −28.92 min, 95% CI: −36.66 to −21.18, *p* < 0.0001), but this subgroup itself displayed high residual heterogeneity (*I*^2^ = 96%), indicating that the cohort estimate is also not a stable, reliable pooled effect. The subgroup difference was statistically significant (*p* < 0.0001).

**Figure 3:**
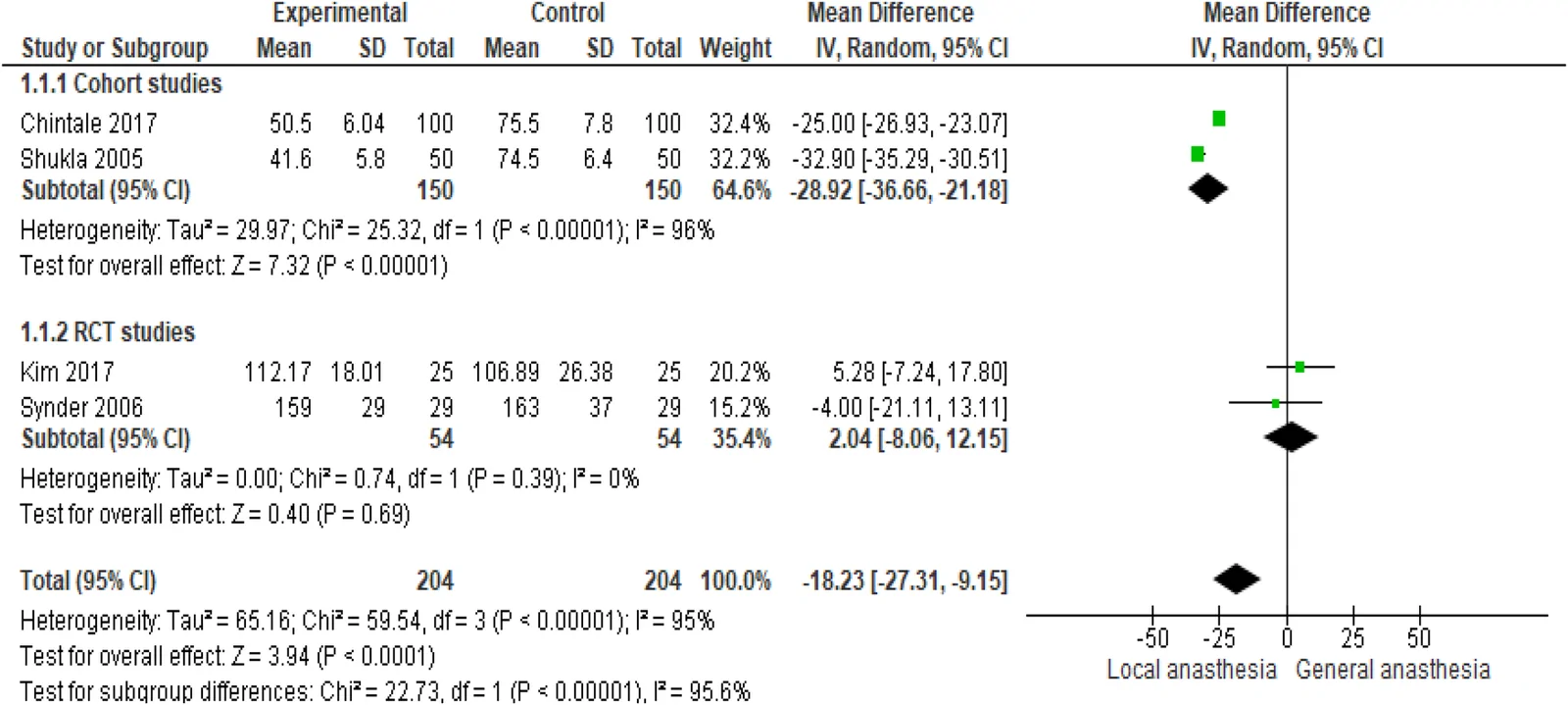
A subgroup analysis of time in OR due to differences in study design.

A leave-one-out sensitivity analysis was performed for the primary outcome by sequentially excluding each of the four contributing studies. The full-model pooled estimate (MD −18.23 min, 95% CI: −27.31 to −9.15, *I*^2^ = 95.0%) was not robust to the exclusion of either cohort study: excluding Shukla (33) shifted the estimate to MD −8.66 min (95% CI: −30.79 to 13.46, *I*^2^ = 92.7%), and excluding Chintale et al. (32) shifted it to MD −11.15 min (95% CI: −39.41 to 17.10, *I*^2^ = 95.5%); in both cases, the 95% CI crossed the null. Excluding either RCT [Kim (38) or Snyder et al. (16)] individually did not change the direction or significance of the pooled estimate (MD −25.31 min, 95% CI: −33.26 to −17.37, *I*^2^ = 93.9%, and MD −20.99 min, 95% CI: −30.44 to −11.55, *I*^2^ = 96.2%, respectively).

### 3.4 Secondary outcomes

Nerve injury: Four studies [Shukla (33), Chintale et al. (32), Kim (38), and Kabuye et al. (8)] reported on nerve injury as a postoperative complication (Table 2). The pooled risk ratio, comprising 316 patients, indicated no significant difference between local and general anesthesia for nerve injury (RR 1.00, 95% CI: 0.45–2.24; Z = 0.00, *p* = 1.00), with negligible heterogeneity [I-squared (heterogeneity statistic, *I*^2^) = 0%; Chi^2^ = 0.30, degrees of freedom (df) = 2, *p* = 0.86] (Figure 4).

**Figure 4:**
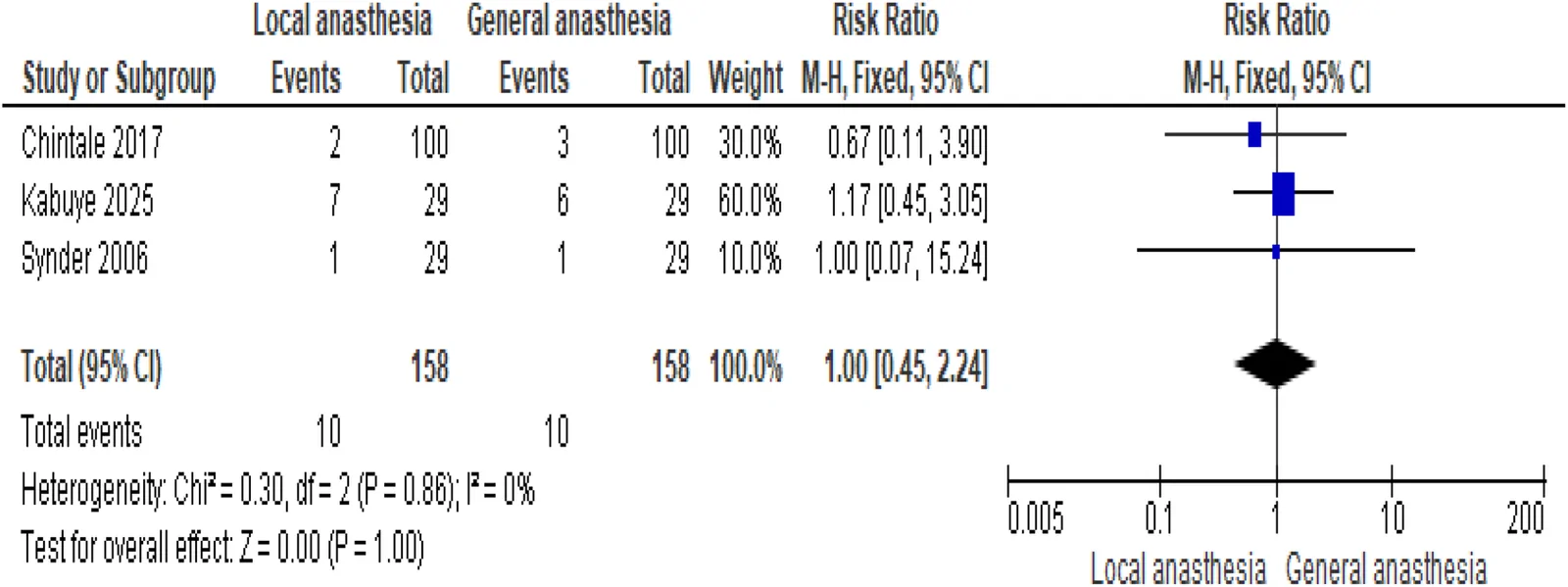
A forest plot of nerve injury.

Respiratory distress: The analysis of respiratory distress pooled 358 patients across three studies [Shukla (33), Kabuye et al. (8), and Chintale et al. (32)]. Three respiratory distress events were recorded (two in the local anesthesia group and one in the general anesthesia group). Given the rarity of events and the presence of zero-event cells in at least one arm, the Peto odds ratio (OR) method was used, as it is recommended by the Cochrane Handbook for outcomes with very low event rates where standard odds ratio or risk ratio estimation is unstable (28). The pooled Peto odds ratio was 1.96 (95% CI: 0.20–19.13), with substantial heterogeneity (*I*^2^ = 63%), suggesting meaningful differences in study design, patient characteristics, or outcome definition across the contributing studies (Figure 5).

**Figure 5:**
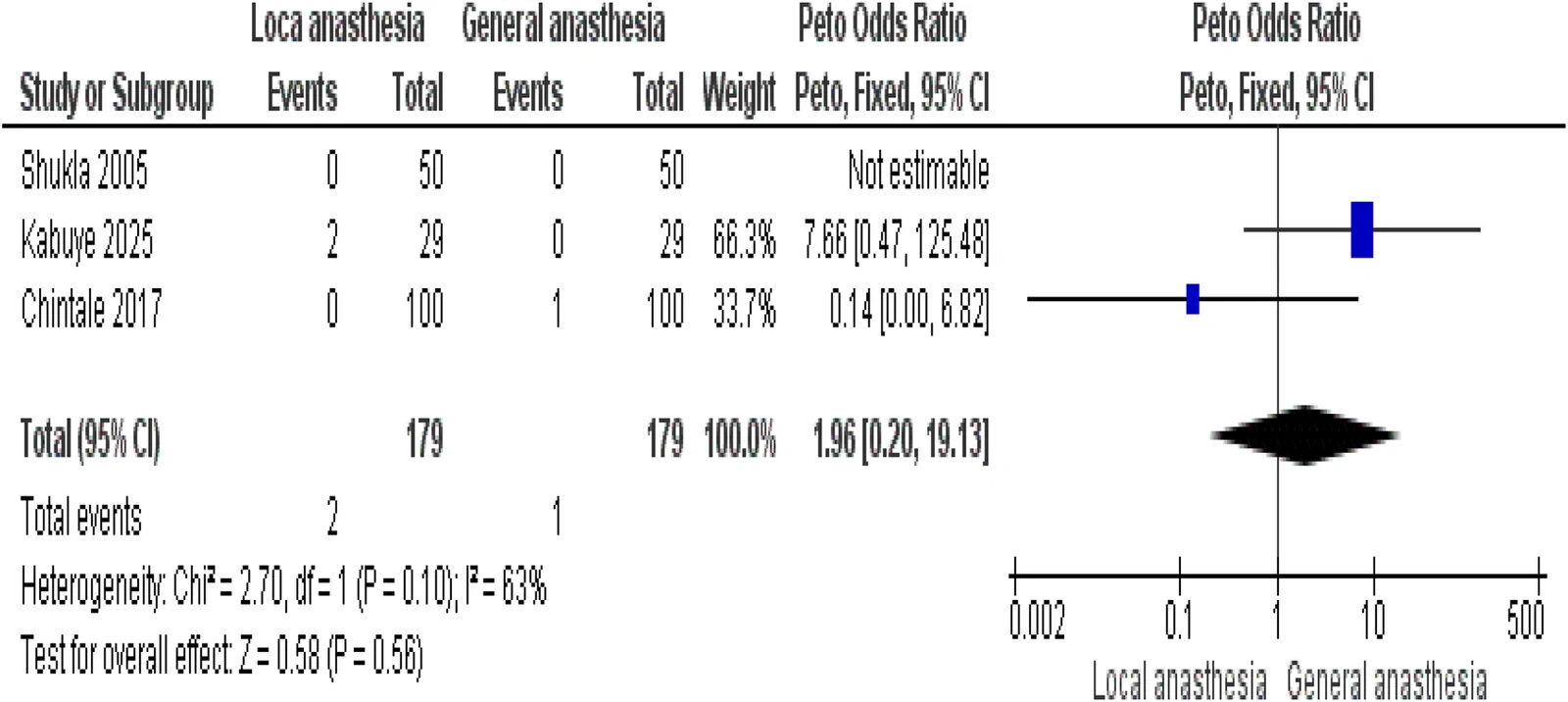
A forest plot of respiratory distress.

Cost: The studies by Chintale et al. (32) and Shukla (33) report the mean overall cost from admission to discharge for local anesthesia [Rs. 2,500 (US $38.42) and Rs. 2,189.32 (US $49.76), respectively] and general anesthesia [Rs. 5,500 (US $84.52) and Rs. 5,520.00 (US $125.45), respectively]; USD equivalents were calculated using the average annual exchange rate for the publication year of each study. The Snyder et al. study reports a hospital cost saving of $315 per patient with local anesthesia with monitored anesthesia care (LA-MAC).

Hypoparathyroidism: Shukla (33) and Snyder et al. (16) reported no cases in the local anesthesia group and one case in the general anesthesia group in Snyder's study (50 and 29 patients per group, respectively). Chintale et al. (32), Kim (38), and Kabuye et al. (8) did not report data on this outcome.

Satisfaction level: Based on the provided sources, patient satisfaction levels were assessed in three of the five studies: Snyder et al. (16), Kim (38), and Kabuye et al. (8). In the Kim et al. study, using a 0–10 visual analog scale on postoperative day 1, there was no significant difference in mean procedure satisfaction scores between the LA-MAC group (7.91 ± 1.90) and the GA group (8.05 ± 1.97). Similarly, the Snyder et al. study, assessing satisfaction at 30 days using a 1–5 scale, found no significant differences in patient satisfaction with anesthesia (*p* = 0.57) or surgical experience (*p* = 0.70) between LA-MAC and GA groups, with 93% of the LA-MAC group and 89% of the GA group reporting satisfaction with anesthesia. The Kabuye et al. study, using a 5-point Likert scale at 30 days, also reported no significant difference in median Likert scores (*p* = 0.442) between the two groups, with the median score being 4 (satisfied) for both GA and LA groups. Across these studies, a high percentage of patients in both local anesthesia groups (with or without MAC) and general anesthesia groups reported that they would recommend the same anesthesia management to others, ranging from 89.7% to 96.6%.

Hospital stay duration: Hospital stay duration was quantitatively assessed in the Chintale et al. (32) and Shukla (33) study, while the Snyder et al. study focused on time to same-day discharge and hospital admission rates. In the Chintale study, the mean hospital stay was 40.26 h for the LA group compared with 75.06 h for the GA group, with LA associated with reduced costs due to shorter stays. Similarly, the Shukla study reported a mean hospital stay of 39.26 h (SD 17.41) for the LA group and 71.06 h (SD 19.36) for the GA group, a statistically significant difference (*p* < 0.05), with 26% of the LA group discharged within 24 h, further contributing to cost savings. The Snyder et al. study, designed for an outpatient setting, found that the LA-MAC group had a significantly shorter time to same-day discharge (165 ± 92 min) compared with the GA group (229 ± 112 min) (*p* = 0.02), with LA-MAC largely eliminating the need for postanesthesia care unit (PACU) time, facilitating earlier recovery. Taken separately, inpatient hospital stay duration was significantly shorter with local anesthesia in both studies that assessed it [Chintale et al. (32) and Shukla (33)], while time to same-day discharge in the outpatient setting studied by Snyder et al. was also significantly shorter with LA-MAC; as these are related but distinct endpoints, measured in different care settings, they should not be interpreted as a single collective effect.

### 3.5 Quality assessment

The ROB assessment for the studies included in this meta-analysis was conducted using two distinct tools tailored to the study designs. For the two randomized controlled trials (Kim and Snyder et al.), the Cochrane RoB 2 tool was applied, evaluating five domains: randomization process, deviations from intended interventions, missing outcome data, measurement of the outcome, and selection of the reported result. Both RCTs demonstrated a moderate to high risk of bias. For the three cohort studies, the ROBINS-I tool was used to assess non-randomized studies of interventions across seven domains, including confounding, participant selection, and outcome measurement. These studies generally exhibited a moderate risk of bias, primarily due to challenges with confounding and missing data. The ROB summary and graph (Figures 6, 7) provide a comprehensive overview of these assessments.

**Figure 6:**
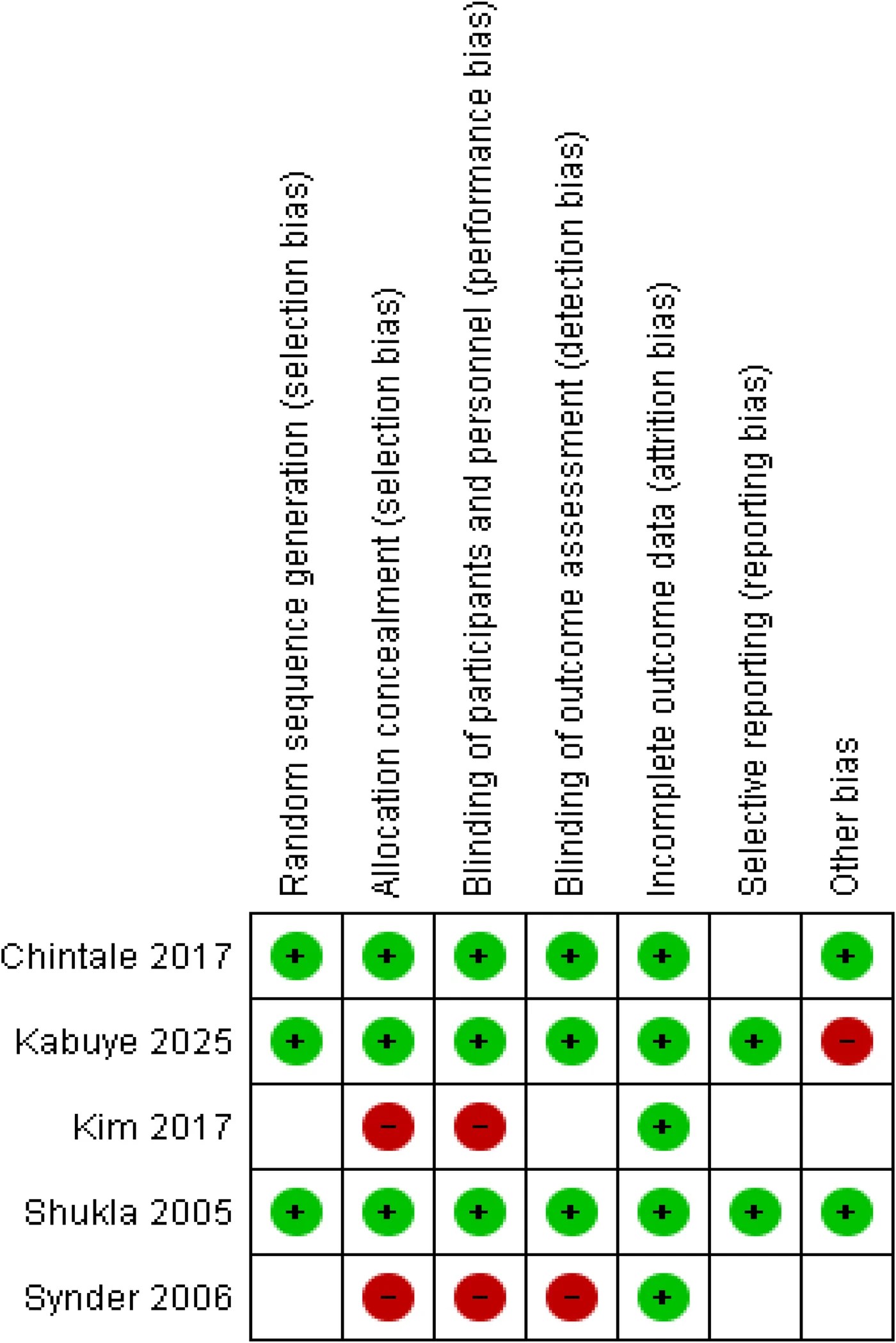
A risk-of-bias summary.

**Figure 7:**
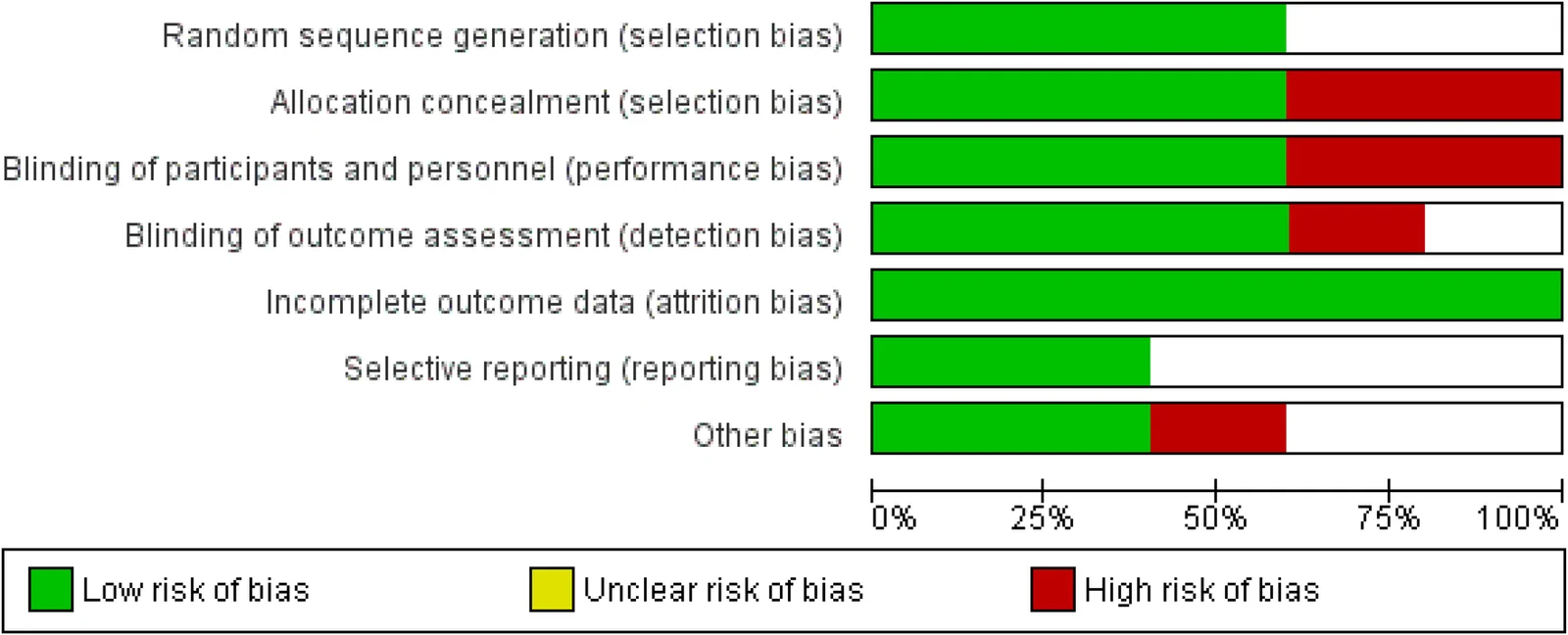
A risk-of-bias graph.

### 3.6 Quality of the evidence

Evidence quality was evaluated according to the GRADE framework for three outcomes of interest. For operating time, data from 408 individuals in two RCTs and two observational studies were assigned as of moderate quality, limited by a minor risk of bias and some inconsistency, while indirectness, imprecision, and publication bias were not considered serious limitations. Nerve injury assessed in 316 individuals from two RCTs and one observational study was rated low quality because of moderate bias risk and serious imprecision; inconsistency and indirectness were not identified as serious problems. The same low quality was assigned to respiratory distress in 358 patients from one RCT and two observational studies, attributed to moderate bias risk, serious imprecision, and a probable publication bias; inconsistency and indirectness posed minor concerns. Overall, the moderate confidence in the operating time results is qualified by minor issues, whereas both the nerve injury and respiratory distress outcomes showed low quality of evidence. A summary of the findings of the GRADE assessment is provided in Table 3.

**Table 3:** A summary of the quality of evidence (GRADE).

Outcome/*N* patients (studies)	Risk of bias	Inconsistency	Indirectness	Imprecision	Publication bias	Overall GRADE quality
Operation time 408 (2 RCT, 2 observational)	Minor concern	Minor concern	Not serious	Not serious	None	Moderate
Nerve injury 316 (1 RCT, 3 observational)	Moderate	Not serious	Not serious	Serious	None	Low
Respiratory distress 358 (0 RCT, 3 observational)	Moderate	Not serious	Not serious	Serious	Possible	Low

GRADE quality ratings: High, very confident the true effect lies close to the estimate; Moderate, moderately confident; Low, limited confidence; Very low, very little confidence. Evidence downgraded based on risk of bias, inconsistency, indirectness, imprecision, and publication bias.

## 4 Discussion

### 4.1 Summary of findings

This systematic review and meta-analysis compared infiltration LA with GA in thyroidectomy across five studies. Compared with GA, LA with MAC was associated with shorter operative time in four of the five included studies, with Kabuye et al. (8) not reporting this outcome. However, this pooled effect was driven disproportionately by the two cohort studies: a subgroup analysis showed no significant difference between LA and GA among the two RCTs alone (MD 2.04 min, 95% CI: −8.06 to 12.15), while the cohort subgroup showed a large, significant difference favoring LA (MD −28.92 min, 95% CI: −36.66 to −21.18) despite substantial heterogeneity within that subgroup (*I*^2^ = 96%). A leave-one-out sensitivity analysis confirmed this instability: excluding either cohort study individually [Shukla (33) or Chintale et al. (32)] rendered the overall pooled effect non-significant. Together, these findings indicate that the apparent operative-time advantage of LA is not a consistent effect across study designs but is instead concentrated in the non-randomized evidence, which is more vulnerable to confounding.

One plausible source of this confounding is disease severity and gland size. Snyder et al. (16) reported a statistically significant difference in resected thyroid weight between the LA and GA groups (26.9 vs. 63.9 g, *p* < 0.05), indicating that patients allocated to GA underwent resection of substantially larger glands. Because gland size and surgical indication are recognized independent determinants of operative duration, and operative time comparisons in this review were not adjusted for either, this baseline imbalance offers a concrete, data-supported explanation for why the cohort studies—which lack randomization to protect against such imbalances—showed a much larger effect than the RCTs. This strengthens, rather than merely qualifies, the case for interpreting the pooled operative-time finding cautiously.

For safety outcomes, LA and GA showed no significant difference in nerve injury (RR 1.00, 95% CI: 0.45–2.24) or respiratory distress (Peto OR 1.96, 95% CI: 0.20–19.13), although certainty of evidence for both was rated low, reflecting serious imprecision from small event numbers. This should not be read as evidence that LA and GA are equally safe—only that no difference was detected with the available evidence, which was underpowered to reliably rule one out.

In addition, this review found consistent evidence that LA was associated with lower costs across all studies reporting cost data, as well as shorter hospital stays, but only inconsistent, design-dependent evidence for a true operative-time advantage, and no evidence of a safety difference in either direction. A more accurate summary is that LA appears to offer perioperative efficiency and cost advantages over GA in select patients, without evidence of increased risk in the safety outcomes assessed, while the certainty of both the effectiveness and safety evidence remains limited.

### 4.2 Comparison with literature

In line with Lo Gerfo and Inabnet et al. (18, 29), we found that LA shortens hospital stays and permits same-day discharge in one of the trials that evaluated this directly [Snyder et al. (16)]. Both of whom reported comparable reductions in length of stay with local or regional approaches; this convergence across an outpatient thyroidectomy series (18) and an evaluation of outpatient feasibility supports hospital stay as the most consistent advantage of LA identified in this review, in contrast to the more fragile operative-time finding (29). Similarly, LA has been shown to shorten postoperative recovery without compromising safety in carotid endarterectomy and endovascular aneurysm repair (30, 31); these results from other surgical specialties lend some external plausibility to a genuine (rather than purely confounded) recovery-time benefit of regional/local approaches, although they cannot resolve the confounding concern specific to our cohort-study evidence above.

On operative time specifically, most individual thyroidectomy studies reported shorter operating times with LA (19, 32, 33), which mirrors our unadjusted pooled result, but our subgroup analysis adds nuance that these individual studies could not provide: when restricted to RCTs only, this advantage disappears, suggesting that differences in surgeon experience, institutional protocol, or patient selection noted elsewhere in the literature as contributing factors may be acting as confounders in the observational evidence specifically, rather than reflecting a true anesthesia-technique effect (34).

On postoperative pain, prior work reporting earlier onset of discomfort under LA without compromising overall recovery quality is consistent with our finding of high patient satisfaction in both groups despite this earlier pain signal—supporting the interpretation that pain timing and overall satisfaction are separable outcomes, and that LA's earlier discomfort does not translate into worse patient-reported experience (13, 35).

On cost, our finding that LA was consistently cheaper across all studies reporting cost data aligns with prior cost-effectiveness literature (8, 19, 35), which similarly attributes savings to reduced anesthesia and hospitalization expenses rather than to differences in surgical technique itself—reinforcing that the cost advantage of LA in this review is plausibly a direct consequence of the shorter hospital stay finding (which, unlike operative time, was consistent across study designs) rather than an independent effect. This benefit is not universal, however: robotic and minimally invasive thyroidectomy approaches remain more costly regardless of anesthesia choice (36, 37), indicating that the cost advantage identified here is specific to conventional thyroidectomy and should not be generalized to newer surgical techniques.

### 4.3 Strengths and limitations

This is the first meta-analysis to compare LA and GA in thyroidectomy, addressing a genuine gap in the literature. The review draws on data from different countries (India, Republic of Korea, Uganda, and the United States), enhancing its relevance across differing healthcare systems, and pools a comprehensive set of perioperative outcomes spanning operative efficiency, complications, cost, and recovery. The study's use of rigorous statistical approaches makes it clear how observational and clinical studies differ, and the impact of design on outcomes. It collected comprehensive clinical data on anesthesia, including operative time, complications, costs, and recovery, which, alongside the other reported outcomes, provides a holistic view of effectiveness. The inclusion of data from other countries, including India, Republic of Korea, and Uganda, enhances the study's applicability for comparison with various healthcare systems and increases its global relevance.

The main limitation of our review is the small number of included studies (*n* = 5), which limits statistical power and precludes formal testing for publication bias. As detailed in Section 4.1, this limitation is compounded by a marked discordance between the two RCTs and three cohort studies for the primary outcome, driven at least in part by an identified baseline imbalance in resected gland weight between treatment arms in one trial—underscoring that the observed operative-time effect should be treated as hypothesis-generating rather than confirmatory. Postoperative pain scores and analgesic use, along with hypocalcemia and postoperative hematoma, were inconsistently or incompletely reported across studies, precluding a formal pooled analysis of these outcomes. Incomplete reporting of thyroid pathology (benign vs. malignant) similarly precluded formal subgroup comparison by disease type, despite malignancy being independently associated with greater surgical complexity and operative time.

A further source of indirectness is variation in age eligibility across the contributing studies: Kabuye et al. (8) restricted enrollment to patients aged 18–65, and Kim (38) required age ≥18, whereas this review applied no upper or lower age limit at the eligibility-criteria level. As a result, the pooled findings may not generalize well to pediatric or very elderly populations, who were likely underrepresented or absent from the contributing evidence base and who may carry different anesthesia risk profiles than the middle-aged adult populations predominantly studied here.

This review was also restricted to studies published in English, which introduces a risk of language bias. Although title/abstract and full-text screening did not identify any Arabic-language studies meeting the remaining eligibility criteria, this does not eliminate the possibility that relevant non-English-language evidence, including Arabic-language studies not indexed or retrievable through our search strategy, was missed by restricting eligibility to English-language publications at the outset.

### 4.4 Implications and future direction

Given the cost and hospital-stay advantages identified—which, unlike the operative-time finding, were consistent across the available evidence—LA may be a reasonable alternative anesthetic approach for selected thyroidectomy candidates, particularly in resource-limited settings where cost-effectiveness and shorter hospital stays meaningfully improve system-level efficiency. Separately, some authors have proposed that intraoperative voice assessment under LA with MAC offers a practical means of monitoring recurrent laryngeal nerve function during surgery (8). We note this as a context-dependent clinical consideration rather than a finding of this review: where intraoperative neuromonitoring (IONM) is available under GA, it provides continuous, proactive electrophysiological nerve monitoring and likely represents the more robust approach to nerve preservation. The voice-assessment advantage of LA is therefore most relevant specifically in settings where IONM is not accessible, such as many resource-limited healthcare environments. In the same settings, this review's cost and hospital-stay findings are most likely to be actionable.

Future research priorities should include large-scale RCTs that adjust for or stratify by thyroid gland size, surgical indication, and pathology; standardized reporting of hypocalcemia, postoperative hematoma, and pain outcomes to permit pooled analysis; explicit reporting and, where possible, subgroup analysis by age group to clarify generalizability across the lifespan; and documentation of IONM use to allow a valid comparison of nerve-injury outcomes between anesthetic approaches.

## 5 Conclusion

This review found that local anesthesia was associated with lower costs and shorter hospital stay compared with general anesthesia in thyroidectomy, findings that were consistent across the studies reporting them. Evidence for a shorter operative time with local anesthesia was less consistent: this effect was concentrated in cohort studies, was not observed in randomized controlled trials, and was not robust to sensitivity analysis and should therefore be interpreted as hypothesis-generating rather than conclusive.

No significant differences in nerve injury or respiratory distress were observed between the two groups, although certainty of this evidence was low given the small number of events; this should not be interpreted as evidence that the two techniques are equally safe. General anesthesia likely remains necessary for more advanced procedures requiring airway control and complete patient immobility, although this review did not directly evaluate outcomes in that population. Future research should prioritize large-scale randomized trials that adjust for thyroid gland size and surgical indication, with standardized reporting of safety outcomes, postoperative pain, and patients' quality of life.

## Data Availability

The original contributions presented in the study are included in the article/Supplementary Material; further inquiries can be directed to the corresponding author.

## Supplementary material

Supplementary Material S1 Full electronic search strategies for PubMed/MEDLINE, Embase, Web of Science, and Scopus.
