# Supplementary material for: Local infiltration anesthesia (anterior field block) vs. general anesthesia for thyroidectomy: effects on operative time, complications, and cost—a GRADE-assessed systematic review and meta-analysis with subgroup analysis
Source: Front Surg. 2026 Sep 30;13:1697921. doi: 10.3389/fsurg.2026.1697921 (PMC13662020; doi:10.3389/fsurg.2026.1697921)
Supplement: Supplementary file 1 [file Table1.docx]

Supplementary Material : Full Electronic Search Strategies

*Local Infiltration Anesthesia (Anterior Field Block) versus General Anesthesia for Thyroidectomy: Effects on Operative Time, Complications, and Cost — A GRADE-Assessed Systematic Review and Meta-Analysis*

Overview

Four electronic databases were searched from inception to August 2025, with the search completed in August 2025: PubMed/MEDLINE, Embase, Web of Science, and Scopus. No restrictions were placed on publication date, geographic region, or healthcare setting. Searches were limited to studies published in the English language.

1. PubMed / MEDLINE

**Platform: PubMed (National Library of Medicine)**

**Search date: [12 July 2025]**

**Full strategy:**

```
#1  "Thyroidectomy"[Mesh] OR thyroidectomy[tiab] OR
    "thyroid surgery"[tiab] OR "thyroid surgeries"[tiab] OR
    "thyroidectomies"[tiab]

#2  "Anesthesia, Local"[Mesh] OR "local anesthesia"[tiab] OR
    "local anaesthesia"[tiab] OR "regional anesthesia"[tiab] OR
    "regional anaesthesia"[tiab] OR "cervical block"[tiab] OR
    "cervical plexus block"[tiab] OR
    "local infiltration anesthesia"[tiab] OR
    "local infiltration anaesthesia"[tiab] OR
    "monitored anesthesia care"[tiab] OR "LA-MAC"[tiab]

#3  "Anesthesia, General"[Mesh] OR "general anesthesia"[tiab]
    OR "general anaesthesia"[tiab]

#4  #1 AND #2 AND #3
```

**Records retrieved: [102]**

2. Embase

**Platform: Embase.com**

**Search date: [12 July 2025]**

**Full strategy:**

```
#1  'thyroidectomy'/exp OR thyroidectomy:ti,ab OR
    'thyroid surgery':ti,ab OR 'thyroid surgeries':ti,ab OR
    thyroidectomies:ti,ab

#2  'local anesthesia'/exp OR 'regional anesthesia'/exp OR
    'local anesthesia':ti,ab OR 'local anaesthesia':ti,ab OR
    'regional anesthesia':ti,ab OR 'regional anaesthesia':ti,ab OR
    'cervical block':ti,ab OR 'cervical plexus block':ti,ab OR
    'local infiltration anesthesia':ti,ab OR
    'monitored anesthesia care'/exp OR
    'monitored anesthesia care':ti,ab OR 'LA-MAC':ti,ab

#3  'general anesthesia'/exp OR 'general anesthesia':ti,ab OR
    'general anaesthesia':ti,ab

#4  #1 AND #2 AND #3
```

**Records retrieved: [122]**

3. Web of Science Core Collection

**Platform: Web of Science (Clarivate), Advanced Search**

**Search date: [12 July 2025]**

**Full strategy:**

```
#1  TS=(thyroidectomy OR "thyroid surgery" OR
        "thyroid surgeries" OR thyroidectomies)

#2  TS=("local anesthesia" OR "local anaesthesia" OR
        "regional anesthesia" OR "regional anaesthesia" OR
        "cervical block" OR "cervical plexus block" OR
        "local infiltration anesthesia" OR
        "local infiltration anaesthesia" OR
        "monitored anesthesia care" OR "LA-MAC")

#3  TS=("general anesthesia" OR "general anaesthesia")

#4  #1 AND #2 AND #3
```

**Records retrieved: [31]**

4. Scopus

**Platform: Scopus (Elsevier), Advanced Search**

**Search date: [12 July 2025]**

**Full strategy:**

```
TITLE-ABS-KEY ( thyroidectomy OR "thyroid surgery" OR
  "thyroid surgeries" OR thyroidectomies )
AND
TITLE-ABS-KEY ( "local anesthesia" OR "local anaesthesia" OR
  "regional anesthesia" OR "regional anaesthesia" OR
  "cervical block" OR "cervical plexus block" OR
  "local infiltration anesthesia" OR
  "local infiltration anaesthesia" OR
  "monitored anesthesia care" OR "LA-MAC" )
AND
TITLE-ABS-KEY ( "general anesthesia" OR "general anaesthesia" )
AND
( LIMIT-TO ( LANGUAGE , "English" ) )

Date range: database inception to August 2025
```

**Records retrieved: [1530]**
